# Effect of Iron Ion on Corrosion Behavior of Inconel 625 in High-Temperature Water

**DOI:** 10.1155/2020/9130362

**Published:** 2020-10-21

**Authors:** Huiling Zhou, Yipeng Chen, Yi Sui, Yunfei Lv, Zhiyuan Zhu, Lanlan Yang, Zhen He, Chengtao Li, Kewei Fang

**Affiliations:** ^1^School of Materials Science and Engineering, Jiangsu University of Science and Technology, Zhenjiang 212003, China; ^2^A. O. Smith (China) Water Heater Co., Ltd., Nanjing 210038, China; ^3^Chengde Petroleum College, Chengde 067000, China; ^4^Suzhou Nuclear Power Research Institute, Suzhou 215004, China

## Abstract

The corrosion behavior of an ultralow iron nickel-based alloy Inconel 625 under high-temperature water has been evaluated. The results show that surface oxidation and pitting were the principal corrosion mechanisms of Inconel 625 during the initial immersion period. The surface layer of the oxide film is first Ni-enriched and then Fe-enriched as immersion time increases. The iron ions dissolved from the autoclave could lead to the formation of NiFe_2_O_4_ and have a great influence on the oxidation behavior of Inconel 625. The oxides nucleated by solid-state reactions with selective dissolution of Fe and Ni and then grew up through precipitation of cations from solution.

## 1. Introduction

The structure and composition of oxide film formed in primary water of pressurized water reactors (PWR) play a significant role in the degradation processes of material, which have been a special topic for decades [1–4]. Therefore, the oxidation behaviors of materials in PWR as well as the characteristics of the oxide film have always been the focus of attention [5]. It had been reported that the properties of the oxide films are closely related to the corrosion resistance [6–10]. The corrosion behaviors of nickel-based alloys in high-temperature water have been extensively studied [1, 7, 11–16]. The oxide film developed on nickel-based alloys generally presents a multilayer structure with Ni/Fe-rich outer layer and a chromium-rich inner layer [14, 17–19]. The structure and the chemical compositions of the oxide film are closely related to the corrosion properties of nickel-based alloys [20–22]. A couple of materials and environment relevant factors, including chemical composition, microstructure, and thickness, influence the oxide film characteristics [2, 19, 23–25]. The effect of water chemistry is also a key factor that determines the characteristic of the oxide film [10, 12, 19, 23, 26], part from the microstructure and chemical compositions of the materials.

In addition to the oxidation, pitting corrosion has been frequently observed on the nickel-based alloys such as Alloy 625 and Alloy 718 [2]. The dissolved ions in high-temperature water are usually related to the oxidation process [27–29]. Kritzer et al. investigated the corrosion behavior of Alloy 625 in high-temperature and high-pressure sulfate solutions and found that the metal ion concentrations keep increasing at the beginning of the experiment [1]. Kuang et al. [30] investigated the effect of Ni^2+^ from autoclave material on 304 SS in oxygenated high-temperature water and found the dissolved Ni^2+^ promotes the stability of NiFe_2_O_4_. Behnamian et al. [31] investigated the oxidation behavior of nickel-based alloys in high-temperature water and observed that the alloys containing Mo, Nb, and Ti were susceptible to pitting. Yang et al. [32] investigated the corrosion behavior of nickel-based alloys in temperature water with 6000 ppm NH_4_Cl. They found that the oxide film is Fe-enriched oxide deposits, which were mainly resulted from the dissolving of Fe in the autoclave. So it is of great significance to investigate the effect of exotic metallic ions on the oxidation behavior of materials in high-temperature water.

The aim of this work is to clarify the effect of the dissolved iron ion from a 304L SS autoclave on the corrosion behavior of Inconel 625 in high-temperature water. The effects of iron ion on the corrosion behaviors of Inconel 625 were investigated by optical microscopy (OM), scanning electron microscopy (SEM), X-ray diffraction (XRD), and Raman spectra measurements. At the end, the corrosion mechanism is explained briefly.

## 2. Experimental

**Figure 1:**
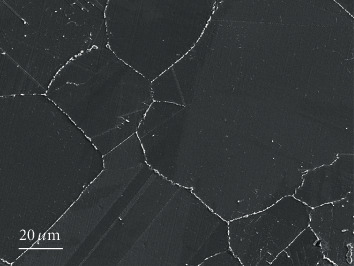
Microstructure of ultralow iron Inconel 625.

The material used in this work is an ultralow iron Inconel 625, the composition of which is listed in Table 1. The microstructure of Inconel 625 has been detailed addressed in literature [33], as is shown in Figure 1. The as-received material was cut into pieces of 30 mm × 20 mm × 3 mm. The samples were mechanically polished to 1000# SiC paper, washed by ultrasound with acetone, and dried with hot air. The weight change of samples was obtained using an electric balance (XS105DU) with an accuracy of 0.1 mg. A corrosion test was conducted in a 2.5 L volume autoclave which is made of 304 L stainless steel (SS). The temperature was controlled at 345°C, under a pressure of 15.5 MPa. The samples were immersed in the autoclave for 100 h, 300 h, 500 h, 700 h, 1000 h, and 1500 h, respectively. In order to ensure the accuracy of the experiment, five parallel samples were adopted.

A scanning electron microscope (XL30-FEG ESEM, FEI, Hillsboro, OR., USA) was applied to observe the morphology evolution of the oxide film. Before SEM observation, the sample surface was coated with a thin Ni layer to avoid the spoliation during sample preparation [34]. Phase constituents were identified by an X-ray diffractometer (XRD, Rigaku Corporation, Tokyo, Japan) with Cu-K_*α*_ radiation with a wavelength of 1.5 Å at 40 kV. The scan range was 20°–90° with a scan rate of 0.1°/s. A laser Raman spectrometer (Renishaw Micro-Raman, UK) with an excitation source of 532 nm wavelength incident laser was applied to identify the oxide composition in the oxide film.

## 3. Results and Discussion

### 3.1. Characteristics of Weight Gain

**Figure 2:**
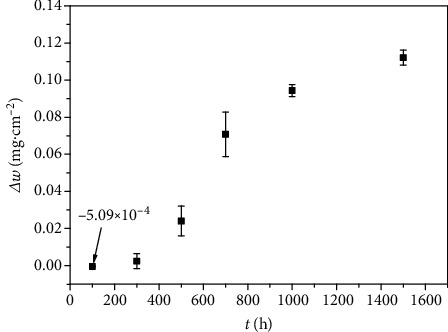
Mass gain of Inconel 625 in high-temperature water.

The result of the weight change with time of Inconel 625 samples in high-temperature water is plotted in Figure 2. The weight gain was negative after immersion in the tested solution for 100 h. The mass loss is 5.09 × 10^–4^ mg·cm^–2^ and this may be due to the simultaneous effects of oxidation and material loss due to pitting [17]. In high-temperature water, the fluctuations of the weight change of the Ni-based alloys could be attributed to the pitting corrosion [2, 35]. A detailed explanation of this phenomenon would be conducted by the surface morphology observation later. However, with the exposure time extending, weight gain increased rapidly when the immersion time was longer than 300 h.

**Figure 3:**
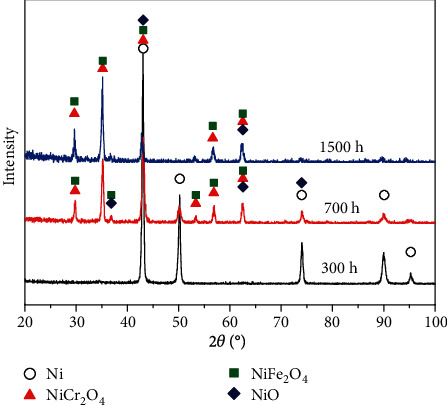
The XRD patterns of oxidation film formed on Inconel 625.

After exposing to high-temperature water for different periods, the oxide products formed on Inconel 625 is detected by XRD, as shown in Figure 3. After 300 h immersion, the samples were only slightly oxidized and the characteristic peaks of oxide could be barely discernable. Then, as the immersion time prolonged, the signals of the base alloy tended to weaken, while those for NiCr_2_O_4_, NiFe_2_O_4_, and NiO gradually dominated.

**Figure 4:**
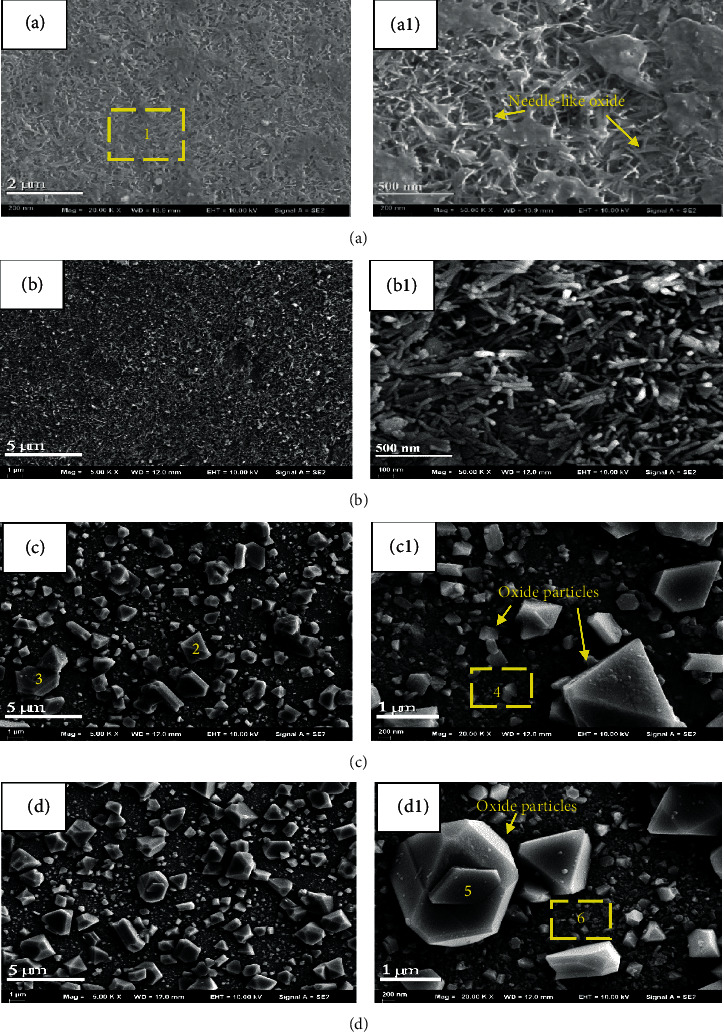
Morphologies of the oxide film formed on Inconel 625: (a, a1) 100 h; (b, b1) 500 h; (c, c1) 1000 h; (d, d1) 1500 h.

The surface morphologies of Inconel 625 after exposing for different periods in high-temperature water are shown in Figure 4. The chemical compositions of the oxide film and oxide particles were characterized using EDS at spots 1–6 and are listed in Table 2. After immersion under high-temperature water for 100 h, a layer of needle-like oxide and polygonal particle oxide formed on the surface of sample, as shown in Figures 4(a) and 4(a1). As the diffusion rates of typical metal ions followed the order of Ni>Cr in Ni-based alloys [12], Ni ions were first detected on the sample surface, which dissolved and precipitated as oxides or hydroxides. The phenomenon is similar to the study of Zhu et al. [15]. From the EDS analysis, it was deduced that these large particles were NiCr_2_O_4_. The consideration was also consistent with the finding of Ziemniak and Hanson [16], who reported that the initial oxidation of Inconel 625 in high-temperature water would create NiO and Ni(Cr,Fe)_2_O_4_.

After immersion in high-temperature water for 500 h, a layer of dense needle-like oxide film appeared, which has been generally observed in Ni-based alloys in PWR water [15, 20, 36]. As displayed in Figure 2(b1), a decrease in density and thickness of needle-like oxide was evident. A layer of dense continuous oxide layer as well as irregularly shaped oxide particles distributed evenly present on the surface, as shown in Figures 4(c) and 4(c1). These irregularly shaped oxide particles varied greatly in size. These oxide particles are iron-oxides according to the EDS analysis presented in Table 2. Combined with the XRD analysis presented in Figure 3, it could infer that the phase is NiFe_2_O_4_. The formation of NiFe_2_O_4_ can be expressed as [37]

(1) $$\begin{array}{c} 2{\text{Fe}}^{2+}+\text{N}{\text{i}}^{2+}+4{\text{H}}_{2}\text{O}\to {\text{NiFe}}_{2}{\text{O}}_{4}+8{\text{H}}^{+}+2\text{e} \end{array}$$

Because the iron content of Inconel 625 in this work was low (0.01%), it was impossible to form iron-containing oxide during the corrosion process. Therefore, it can be deduced that the iron ions may be due to the dissolution of the autoclave material and then deposited on the sample surface [30, 37]. EDS chemical analyses indicated that the oxide film was mainly composed of Ni and Cr oxides with a small amount of Fe, Nb, and Mo, as shown in Table 2. Consequently, it could be drawn that the oxide film was mainly NiO and Ni(Cr,Fe)_2_O_4_. This agreed well with those reported in the literatures for nickel-based alloys [26, 27, 36].

The surface morphology was somewhat similar to that of 1000 h after an immersion period of 1500 h, as shown in Figure 4(d). The irregularly shaped particles developed gradually both in size and in number (Figure 4(d1)). A similar phenomenon had been observed by Clair et al. [27]. The EDS analysis revealed the oxide film as a binary mixture of NiCr_2_O_4_ and NiFe_2_O_4_ [27].

### 3.2. Pitting Corrosion

**Figure 5:**
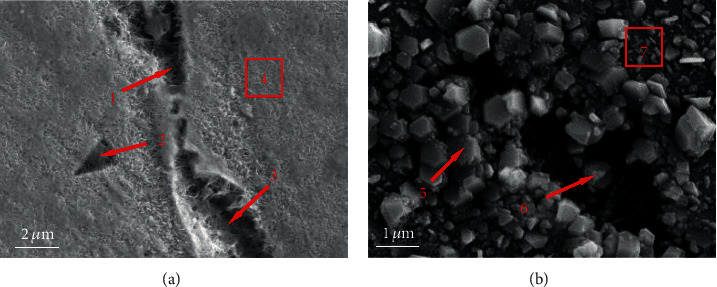
Morphologies of pitting corrosion for Inconel 625 in high-temperature water: (a) 100 h; (b) 700 h.

In addition to the oxidation, pitting corrosion also occurred on the Inconel 625. EDS composition analysis was carried out on the oxide film and corrosion pits at spots 1–7, and the obtained data are presented in Table 3. Pitting corrosion was observed after an immersion period of only 100 h, as shown in Figure 5(a), which was consistent with the results of Behnamian et al. [31] and Yang et al. [32]. The observation strongly supported the result of mass loss presented in Figure 2. In this case, the amount of weight gain caused by the formation of oxide film on the surface was less than the weight loss that is caused by pitting and uniform corrosion. The EDS results suggested that high levels of niobium element accumulated in the corrosion pits. It seemed that the corrosion pits might be determined by inclusions of NbC [31, 38, 39]. In high-temperature water, pitting may be associated with Nb-rich precipitates (likely the *γ*'-phase) [4, 40] and induced by local potential difference between the matrix and the inclusions [2].  Figure 5(b) showed that the irregularly shaped oxide particles distributed evenly on the surface. Base on the EDS analysis presented in Table 3, the oxide particle was the Nb-rich phase, indicating that the occurrence of the Nb-rich phase and then the dissolved Nb^2+^/Nb^3+^ ion form oxide that deposited in the vicinity of the corrosion pit. Solution treatment can reduce the precipitation of the secondary phases, optimize the microstructure, and improve the pitting corrosion resistance of metallic alloys [41–43].

**Figure 6:**
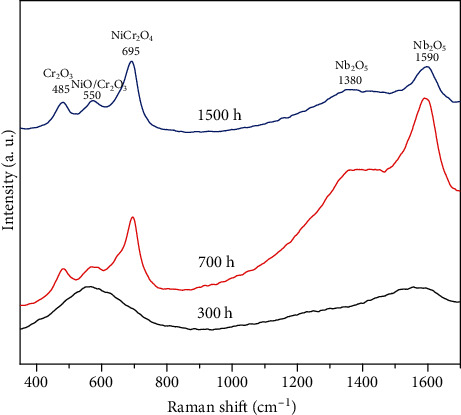
Raman spectra of the oxide films formed on Inconel 625 in high-temperature water.

Figure 6 displays the Raman spectra results of oxide films on Inconel 625. The characteristic peaks located at 485, 550, 695, 1380, and 1590 cm^−1^ indicated that the oxide film mainly consisted of NiO [44], NiCr_2_O_4_ [44], Cr_2_O_3_, and Nb_2_O_5_ [45]. After 300 hours of immersion, the oxide films were composed of NiO and Nb_2_O_5_. The formation of Nb_2_O_5_ mainly resulted from the pitting corrosion of the NbC phase [31, 32]. After 700 hours of immersion, the peaks of Cr_2_O_3_ and NiCr_2_O_4_ gradually appeared. The formation of these continuously dense oxides could protect the alloy effectively. It was reported that NiO was not as stable as the spinel and chromium oxides [2, 34]. Besides, studies showed that element Fe caused solid solution strengthening when added into Ni-based alloy. But others considered that this was due to the low content of oxides produced by iron [17], resulting in the formation of NiCr_2_O_4_ instead of FeCr_2_O_4_. Moreover, the diffusion rate of metal cation Ni^2+^ was much higher than that of Cr^3+^ in oxide film [46]. As a result, the oxide dominated by NiO was formed in the outer layer, while Cr_2_O_3_ and NiCr_2_O_4_ formed in the inner layer [17].

**Figure 7:**
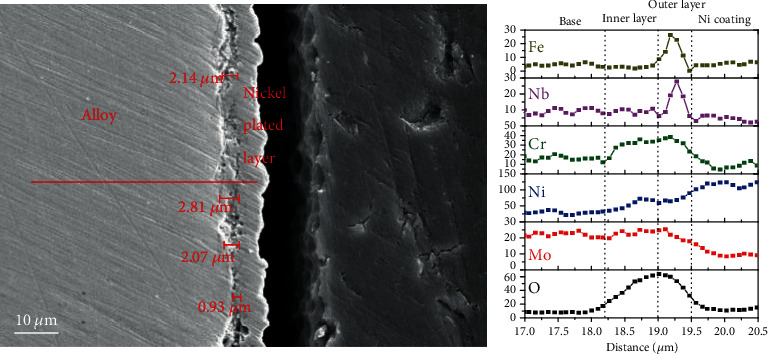
Cross-section of Inconel 625 after corrosion oxidation 1500 h and the element distribution line scan.

The cross-section of Inconel 625 exposed to high-temperature water for 1500 h is shown in Figure 7. The average thickness of the oxide film is about 1.99 *μ*m. The EDS result of line scanning element distribution is presented in Figure 7(b). It is clear that the oxide film shows a double-layer structure and the thickness of the inner layer is slightly thicker than that of the outer layer. Thus, it is inferred that the inner oxide film is mainly composed of NiCr_2_O_4_. In addition, elements Nb and Fe were on the surface of the oxide layer. NiFe_2_O_4_ formed by iron deposition and Nb oxides formed by NbC detachment are on the surface of the oxide films. Although the alloy was oxidized for 1500 h in high-temperature water, the matrix oxide film formed into a continuously dense layer which was still thin, indicating an excellent corrosion resistance to the alloy.

### 3.3. Corrosion Mechanism

**Figure 8:**
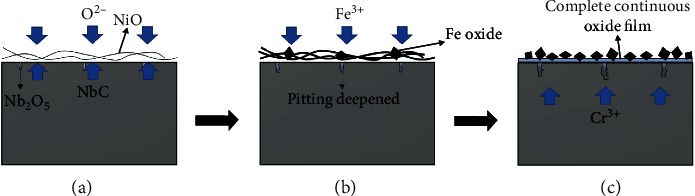
Corrosion mechanism of Inconel 625 in high-temperature water.

Inconel 625 exhibited two oxide layers which were coincident with the SEM observation of the surface. Attributes of dense and uniform are showed by the inner layer and randomly oriented grains with rather different compositions that make up the outer layer [33]. The oxide film consists of a combination of NiCr_2_O_4_, NiFe_2_O, and NiO. On the basis of the evolution of the corrosion process, it is clearly deduced that the elements of 304L SS (autoclave) could be dissolved into the high-temperature water during the immersion test. The dissolved Fe^2+^ could form relatively large, randomly oriented grains of oxide which appeared as polyhedron crystals with sharp edges towards the outermost surface, as shown in Figure 4(c), giving rise to the emerging oxide diffraction peaks. As showed in Table 2 and Figure 4(c), the composition of oxides primarily corresponded to NiFe_2_O_4_, and the matrix oxide film was composed of NiCr_2_O_4_ and NiO according to the diffraction peak. After 1500 h, oxide films adequately covered the alloy surface (Figure 4(d)), leading to the increase of NiCr_2_O_4_ and NiFe_2_O_4_ diffraction peaks. Under these conditions, NiO interacted with the chromium ions diffused from the alloy matrix and the iron ions dissolved from the autoclave, thus giving rise to the formation of NiCr_2_O_4_ and NiFe_2_O_4_, respectively. It should be noted that the protective effect brought by NiCr_2_O_4_ [38] increases along with the increasing corrosion time, as well as the oxidation resistance. This well explains the weight gain trend observed in Figure 1.  Figure 8 shows the corrosion mechanism of Inconel 625 in high-temperature water. The outer layer, consisting of relatively large, randomly oriented grains of Ni(Fe,Cr)_2_O_4_, is formed by iron dissolve from 304L SS (autoclave) and outward diffusion of ion from the base, which grows on the original surface of the sample.

## 4. Conclusion

Inconel 625 in 345°C/15.5 MPa water of 304 SS autoclave resulted in pitting corrosion on the surface in the early stage of corrosion oxidation, and Nb_2_O_5_ was deposited in the pit. NiO, Cr_2_O_3_ was formed as the inner layer, with the progress of corrosion oxidation. Sparse oxide grows preferentially and completely covered the matrix. Fe^2+^ in 304 SS autoclave is dissolved in electrolyte and forms the outer layer composed of scattered crystallites, which is granular and very sensitive to the oxidation conditions. In the later stage of corrosion and oxidation, Ni(Fe,Cr)_2_O_4_ was formed by outward diffusion of Fe and Cr, which is also the components of the outer layer.

## Figures and Tables

**Table 1:** Chemical composition of the tested Inconel 625 (wt%).

C	Cr	Mo	Co	Nb	Fe	Al	Ti	Si	P	S	Ni
0.05	22.4	9.5	0.03	3.66	0.01	0.24	0.23	0.04	0.002	0.001	Bal.

**Table 2:** The EDS chemical compositions (wt%) of the tested spots presented in Figure 4.

	Ni	Cr	Mo	Nb	Fe	O
1	69.60	18.73	6.15	—	—	5.52
2	12.71	3.44	—	—	59.94	23.91
3	14.87	3.39	—	—	56.17	25.57
4	40.41	20.05	8.13	3.27	15.41	12.71
5	20.91	0.96	—	—	51.97	26.16
6	45.99	18.69	6.99	—	16.72	11.62

**Table 3:** EDS chemical composition of the tested spots presented in Figure 5 (wt%).

Area	Ni	Cr	Mo	Nb	Fe	O
1	39.50	13.84	3.85	31.43	—	11.38
2	22.91	6.39	1.33	54.45	—	14.92
3	22.17	7.79	6.59	1.88	—	7.67
4	69.62	21.55	3.82	—	—	5.00
5	29.16	—	—	21.03	25.82	23.99
6	—	—	—	81.88	—	18.12
7	54.89	20.59	6.61	—	8.81	9.09

## Data Availability

The data used to support the findings of this study are available from the corresponding author upon request.
